# Recombinant *Salmonella enterica* Serovar Typhimurium as a Vaccine Vector for HIV-1 Gag

**DOI:** 10.3390/v5092062

**Published:** 2013-08-28

**Authors:** Nyasha Chin’ombe

**Affiliations:** Department of Medical Microbiology, University of Zimbabwe, Avondale A178, Harare, Zimbabwe; E-Mail: nyasha.chinombe@gmail.com; Tel.: +263-4-791-631 ext. 2419; Fax: +263-4-792-245; Division of Medical Virology, University of Cape Town, Observatory 7925, Cape Town, South Africa

**Keywords:** *Salmonella*, vaccine, vector, HIV-1 Gag, immune response

## Abstract

The HIV/AIDS epidemic remains a global health problem, especially in Sub-Saharan Africa. An effective HIV-1 vaccine is therefore badly required to mitigate this ever-expanding problem. Since HIV-1 infects its host through the mucosal surface, a vaccine for the virus needs to trigger mucosal as well as systemic immune responses. Oral, attenuated recombinant *Salmonella* vaccines offer this potential of delivering HIV-1 antigens to both the mucosal and systemic compartments of the immune system. So far, a number of pre-clinical studies have been performed, in which HIV-1 Gag, a highly conserved viral antigen possessing both T- and B-cell epitopes, was successfully delivered by recombinant *Salmonella* vaccines and, in most cases, induced HIV-specific immune responses. In this review, the potential use of *Salmonella enterica* serovar Typhimurium as a live vaccine vector for HIV-1 Gag is explored.

## 1. Introduction to *Salmonella* Bacterium

The Salmonellae belong to the Enterobacteriaceae family of enteric gram-negative and facultatively anaerobic bacteria [1,2]. They cause disease symptoms that range from gastroenteritis to severe systemic fevers in several animals such as mammals, birds and reptiles [3]. The genus *Salmonella* is divided into two species, *Salmonella enterica* and *Salmonella bongori*. *Salmonella enterica* is further classified into six subspecies [4]. *S. typhi* and *S. typhimurium* are now classified as *Salmonella enterica* subspecies enterica serovar Typhi and *Salmonella enterica* subspecies enterica serovar Typhimurium or simply referred to as *Salmonella* *enterica* serovar Typhi and *Salmonella* *enterica* serovar Typhimurium, respectively. Most serovars of *Salmonella* are host adapted, while others are host-restricted [5]. An example of each of these are *S. enterica* serovar Typhi and *S. enterica* serovar Typhimurium, which cause typhoid in humans and mice, respectively. *S. enterica* serovar Typhimurium causes mild gastroeroentritis in humans, but may cause fatal typhoid in mice*. S. enterica* serovar Typhi is host-restricted to humans where it causes typhoid but does not infect mice or other animals. *S. enterica* serovar Typhimurium has therefore been used as a mouse model for the human typhoid disease and is suitable for use in preclinical studies involving the development of recombinant *Salmonella* vaccine vectors. 

Infection of host by *Salmonella* occurs mainly through the oral/gastric route after consumption of contaminated food or water. The invasion of the mucosa-associated lymphoid tissue (MALT) by the bacteria occurs mainly via the M cells [6]. The bacteria start replicating in the Peyers patches of the intestines and eventually disseminate to systemic organs such as the spleen and liver through the mesenteric lymph nodes [7]. Some strains of *Salmonella*, which are less virulent or which are genetically attenuated, are unable to cause severe systemic symptoms because of reduced capacity to invade, replicate, and spread. The ability of such attenuated *Salmonella* to colonize and invade the MALT and spread to distal sites such as liver and spleen with limited symptoms and disease makes them potential candidates for delivery of vaccines of mucosal pathogens such HIV [8].

## 2. Immune Responses to *Salmonella* Infection

*Salmonella* infection can trigger both the innate and adaptive arms of the host immune system [9,10,11]. The innate immune system is provoked by the host’s recognition of *Salmonella* pathogen-associated molecular patterns (PAMPs), such as bacterial lipopolysaccharides, flagellin, polycytosine guanine (CpG) motifs (bacterial DNA), and peptidoglycan [12]. These bacterial PAMPs are recognized by host pattern-recognition receptors such as toll-like receptors (TLRs), thereby facilitating immunostimulation of the innate system [13]. TLR4 recognizes bacterial LPS and is expressed in the host intestinal epithelial cells [14]. TLR5 is associated with the recognition of bacterial flagella. Defensins produced by phagocytes have been implicated in the killing of *Salmonella* soon after infection [15]. Cytokines such as tumor necrosis factor-alpha (TNF-α) and interferon-gamma (IFN-γ) produced by the cells of the innate immune system, such as dendritic cells, have antimicrobial activities and are also involved in control of *Salmonella* infection [16]. The innate immune system can, therefore, control early *Salmonella* infection by phagocytosis and production of antimicrobial molecules. However, the innate immunity alone cannot clear virulent *Salmonella* infection without the assistance of the adaptive immune system [17].

CD4^+^ and CD8^+^ T lymphocytes are crucial for protective immune responses against many intracellular bacterial pathogens such as *Salmonella* [11,18]. In most cases, these cells are critical for sterilizing immunity against bacterial infection [19,20]. The major histocompatibilty complex class I and class II antigen-processing pathways are responsible for the activation of antigen-specific CD8^+^ and CD4^+^ T lymphocytes, respectively [21,22]. CD8^+^ T cells always recognize peptides bound to the MHC class I molecules while CD4^+^ T cells recognize peptides bound to the MHC class II molecules. CD4^+^ and CD8^+^ T cell responses target most of the *Salmonella* antigens such as protein antigens, porins, flagellin, pilin, LPS, and Vi surface polysaccharides [23]. After phagocytosis by phagocytes, the *Salmonella* bacteria replicate in the *Salmonella*-containing vacuoles (SCVs) [24]. *Salmonella* antigens or peptides are therefore predominantly presented by MHC class II molecules to the CD4^+^ T cells [25]. The generation of CD4^+^ T cell responses directed against epitopes of the natural *Salmonella* FliC antigen has been observed in vaccinated mice [26]. It was also shown that macrophages and dendritic cells infected with *Salmonella* could process and present FliC epitopes, resulting in stimulation of antigen-specific CD4^+^ T cell proliferation and IFN-γ secretion [26]. It has also been shown that MHC class II knockout and CD4 knockout mice are highly susceptible to *Salmonella*, underlining the critical role of CD4^+^ T cell responses in protection [27]. IFN-γ knockout mice have also been shown to fail to be susceptible to disseminated septicaemia after *Salmonella* infection [28].

The role of CD8^+^ T cells in controlling intracellular pathogens such as *Salmonella* is also well recognized [29,30]. Since *Salmonella* bacterium resides and replicates in the SCVs, it is not obvious how the processing and presentation of exogenous antigens by the classical MHC class I pathway for induction of CD8^+^ T cell responses will occur. However, recent studies have recorded the induction of *Salmonella*-specific CD8^+^ T cells after bacterial infection in humans and mice [20,31,32,33]. It was shown that a CD8^+^ epitope derived from *Salmonella* HSP-60 could be processed and presented to CD8^+^ T cells [20]. It was further shown that *Salmonella* vaccine vectors could elicit antigen-specific CD8^+^ T cell responses in mice [34]. The mechanisms by which exogenous antigens (from the SCVs) are cross-presented by the MHC class I molecules to give rise to CD8^+^ T cell responses are not clear. It has, however, been suggested that apoptotic cells infected with antigens could be an important source for cross-priming in such situations [35]. *Salmonella*-infected cells undergo bacterial-induced apoptosis and the apoptic blebs could be the main sources of antigens for the generation of *Salmonella*-specific CD8^+^ T cells [36,37]. Bystander dendritic cells have been suggested to be the antigen-presenting cells that engulf the *Salmonella*-infected apoptotic cells for induction of CD8^+^ T cells [37,38]. Dendritic cells are also capable of processing and cross-presenting exogenous antigens for induction of CD8^+^ T cell responses [33,39,40]. Despite our poor understanding of cross-presentation, the fact that *Salmonella* induce CD8^+^ T cell immune responses means that the attenuated bacteria can be usefully exploited as vaccine vectors for HIV from which protection also requires the induction of such immune responses.

*Salmonella* infection further elicites humoral immune responses, which contributes to successful control of bacterial infection [41,42]. Mice challenged with *Salmonella* elicit antibody responses to several antigens such as LPS, flagella, fimbriae, porins, lipoproteins, heat-shock proteins, and other bacterial proteins such as outer membrane proteins [43,44]. Although antibodies are produced against several *Salmonella* antigens, their general role in preventing or controlling infection is unclear. Studies in humans have shown that high antibody titres, specific to *Salmonella* surface antigens, correlated with protection against bacterial infection [45]. Passive transfer of immune serum or B cells has been found to be protective against *Salmonella* infection in mice [46]. B-cell deficient mice have increased susceptibility to *Salmonella* infection [47,48]. Recent work has also shown that *Salmonella* porins induce lifelong bactericidal antibody memory responses in mice [44]. Attenuated *Salmonella* vaccines can therefore be used as recombinant vectors that are capable of inducing foreign antigen-specific antibody responses.

Pathogens such as *Salmonella*, which invade at mucosal surfaces, provoke mucosal and systemic immune responses. At mucosal compartments, the expected B cell immunity comprises mainly secretory immunoglobulin A (s-IgA), while serum IgG immune response is expected in the systemic compartments [49,50,51]. Experimental evidence shows that mucosal secretory IgA correlates with resistance to bacterial infection [52,53,54]. The two types of antibodies (IgA and IgG) potentially neutralize the pathogens and control infection in the mucosal and systemic compartments respectively. T-cell-mediated immune responses can also control *Salmonella* infection at both the mucosal and systemic compartments. It has been documented that T cells produced at one mucosal surface are capable of homing and offer protection at other mucosal surfaces [55,56]. This is one of the key advantages of oral vaccines such as attenuated *Salmonella* and can therefore potentially be used as vaccines for HIV, which is also a mucosal pathogen.

## 3. Attenuated *Salmonella* Vaccines

It is possible to attenuate virulent *Salmonella* genetically. Currently, the genes which have been targeted for attenuation and generation of *Salmonella* vaccines, are those involved in biosynthesis, regulation, and virulence pathways [57,58]. Methods such as signature-tagged mutagenesis (STM) can now be used to completely delete single or multiple genes so as to guarantee complete safety of the vaccines in humans or animals. A number of attenuated *Salmonella* vaccine candidates for prevention of typhoid fever have already been developed. Ty21a was the first attenuated typhoid fever vaccine and was generated by chemical and UV mutagenesis of the *galE* gene [59,60]. It was shown that Ty21a induced systemic CD4^+^ T cells secreting IFN-γ and antibody responses in vaccinated individuals [61]. Human trials in Egypt also showed protective efficacy of 96% and the period of protection was three years after vaccination with Ty21a [62]. Recent studies have further confirmed that immunization of humans with Ty21a induced both CD4^+^ and CD8^+^ T-cell responses in peripheral blood, together with mucosal IgA and serum IgG antibody responses [63]. The study demonstrated that despite being attenuated, Ty21a vaccine could still induce immune responses. There are still other live attenuated vaccines under development. Examples of these live attenuated *Salmonella* vaccines with known genetic mutations include *aro* mutants, such as *Salmonella* *enterica* serovar Typhi CVD906, and CVD908, *cya/cry* mutants, such as *Salmonella* *enterica* serovar Typhi Chi3927, and *PhoP/Q* mutants, such as *Salmonella* *enterica* serovar Typhi Ty800 [64,65,66,67]. Humans vaccinated with CVD906 have developed strong immune responses against LPS, although there were some adverse symptoms such as fever and bacteraemia in some vaccinees [68]. Studies with CVD908 showed that the vaccine was highly immunogenic, with induction of *Salmonella* LPS-specific IgG and IgA antibodies [69]. CVD 908-htrA vaccine was shown to induce both CD4^+^ and CD8^+^ T cell responses in vaccinated volunteers [70]. Ty800 (aroA phoP mutant) was shown to be safe and immunogenic by inducing IgA and serum IgG antibody responses in Phase I clinical trials [71]. Recent studies of another oral typhoid vaccine, M01ZH09, which has non-reverting mutations in *aroC* and *ssaV* genes, have shown that it is well tolerated and very immunogenic, even after a single vaccination [72,73]. All these attenuated *Salmonella* vaccines have the potential to be harnessed as vaccine vectors for HIV and other pathogens.

## 4. Advantages of Using *Salmonella* as an HIV Vaccine Vector

The ability of attenuated *Salmonella* vaccines to induce both cellular and humoral immune responses at both mucosal and systematic compartments makes them good candidates for use in delivery of heterologous antigens. These live attenuated *Salmonella* vaccines have several advantages for use as delivery systems, especially of mucosal pathogens. They mimic the natural infection of most mucosal pathogens such as HIV-1, which infect their host through mucosal surfaces. They are intracellular pathogens, which are capable of surviving and replicating inside antigen-presenting cells (dendritic cells and macrophages) [74]. This facilitates the continual processing and presentation of the foreign antigens to the immune system. The vaccines are relatively inexpensive to produce or manufacture for large-scale mass-immunizations. There are now non-reverting live attenuated *Salmonella* strains developed using modern technologies in genetic engineering. These mutants cannot revert to wild-type and can therefore be safe for use in humans. This property may make it possible for *Salmonella* vaccines to be used even for patients infected with HIV-1 and are immunocompromised. The bacterial vaccines are easily treatable with antibiotics should adverse effects occur during immunizations. The oral route is more practical, socially acceptable, and reliable than other routes of vaccine administration. The oral (mucosal) vaccination also results in induction of both mucosal and systemic immune responses, unlike systemic vaccination, which does not normally elicit mucosal immunity. *Salmonella* bacterium can hold large amount of foreign DNA (large multivalent antigen capacity) and, therefore, one or more foreign antigens can be delivered. In addition, the molecular tools and techniques developed over the years for genetic manipulation of *E. coli* can easily be applied to *Salmonella* vaccine manipulation. All these advantages make *Salmonella* vaccines attractive for use as recombinant vectors for HIV-1. However, despite all the advantages mentioned above, live attenuated *Salmonella* may have few potential pitfalls as vaccine vectors. The problems that may be encountered include (i) high instability, especially when high copy number plasmids are used; (ii) loss of plasmid during cell division over generations; (iii) poor immunogenicity if the antigens are not expressed at high levels; (iv) metabolic burden to the *Salmonella* vector if the foreign antigens are expressed at very high levels; (v) post-translational cellular proteolytic degradation of the foreign antigens; and (vi) no post-translational modification of expressed proteins. Despite these problems, *Salmonella* vaccines have already been used to deliver a number of viral, bacterial, parasitic, and other antigens to the immune system. Vaccination of animals or human volunteers with some of the recombinant *Salmonella* has resulted in immune responses directed against the heterologous antigens. Most of the preclinical studies have been conducted using attenuated *Salmonella* *enterica* serovar Typhimurium, which is an infection model in mice and for *Salmonella* *enterica* serovar Typhi infection in humans.

## 5. Rationale of Targeting HIV-1 Gag as a Vaccine Antigen

The HIV/AIDS epidemic is a global health issue and an effective vaccine is required to mitigate the problem. Most of the HIV-1 vaccines currently under development are based on the *gag* gene. There are a number of reasons why the HIV-1 *gag* gene has been selected for vaccine development. HIV-1 Gag is one of the most highly conserved structural antigens and can, therefore, be targeted for the development of a vaccine for diverse HIV-1 subtypes [75,76]. In natural infection, HIV-1 Gag-specific CD8^+^ T cells play an important role in controlling primary HIV-1 viremia, thereby slowing disease progression over time [77,78,79,80,81,82]. Furthermore, Gag-specific CD8^+^ T cell responses have broad cross-reactivity for diverse HIV-1 subtypes and strains [83,84]. Gag also contains a number of immunodominant T- and B-cell epitopes, which are conserved among HIV-1 subtypes and strains [85,86,87]. Thus, targeting the conserved HIV-1 proteins such as Gag for development of *Salmonella*-based vaccines is logical.

## 6. *Salmonella* Vaccine Vectors Expressing HIV-1 Gag

A number of studies have so far been done to deliver HIV-1 Gag to the immune system by using recombinant *Salmonella* expressing the antigen. Most of these studies have used prokaryotic expression plasmids, with the HIV-1 *gag* gene cloned in these systems. Thus far, the immunogenicity studies have been done mainly in mice and with only one study reaching human trials. 

Our research group was recently investigating the potential use of *Salmonella* as a vaccine vector for HIV-1 antigens. We successful constructed a recombinant *Salmonella* overexpressing codon-optimized HIV-1 Subtype C Gag in the bacterial cytoplasm [88]. When groups of BALB/c mice were orally vaccinated three times, systematic HIV-1 Gag-specific CD4^+^ Th1 and Th2 cytokine responses were provoked [88]. For Th1 responses, both HIV-1-specific interferon-gamma and tumor necrosis factor-alpha cytokines were elicited and for the Th2 responses, interleukin-4 and interleukin-5 cytokine responses were elicited [88]. Evaluation of humoral responses in these mice showed that HIV-1 Gag-specific IgG1 (Th1) and IgG2a (Th2) were also produced [88]. The vaccinated mice did not elicit Gag-specific CD8^+^ T cell response. This was perhaps due to the fact that the Gag antigen was expressed only as bacterial inclusion bodies, which are particulate aggregations and are likely only to be presented well to the CD4^+^ T cells. We were, however, able to show that the recombinant *Salmonella* that overexpressed GFP as a soluble antigen could induce GFP-specific CD8^+^ T cell responses in vaccinated mice [89].

Secretion of HIV-1 Gag antigens by a recombinant *Salmonella* is one of the strategies to improve immune responses. A study by Bachtiar and colleagues showed that a recombinant *Salmonella* vaccine expressing of HIV-Gag (p24) in a prokaryotic expression vector under the control of a hemolysin secretorial signal of *E. coli* could induce Gag-specific humoral and T cell responses in orally vaccinated mice [90]. In the same study, a recombinant *Salmonella* carrying an HIV-1 Gag DNA vaccine was also shown to be immunogenic in mice [90]. The results from this study therefore confirmed that recombinant *Salmonella* vaccines could deliver HIV-1 antigens to the immune system to induce HIV-1-specific immune responses. In another study, HIV-1 Gag fused to a *Salmonella* Type III secretion system SopE protein was secreted from a recombinant *Salmonella* vaccine vector [91]. When human volunteers were vaccinated using this vector, 83% (15/18) elicited *Salmonella*-specific mucosal immune responses [91]. However, none of the subjects elicited HIV-1 Gag-specific humoral and cellular responses [91]. The lack of HIV-1 Gag-specific response could be a result of a single vaccination or the use of *Salmonella enterica* serovar Typhimurium instead of *Salmonella enterica* serovar Typhi.

Genes for foreign antigens can also be expressed from *Salmonella* chromosome instead of from an expression plasmid. This increases stability of the bacterial vector, but has a drawback of low gene dosage [92]. In a study, *Salmonella enterica* serovar Typhi was used to express HIV-1 Gag from the chromosome [92]. Mice vaccinated intranasally elicited Gag-specific cytotoxic T lymphocyte responses in the spleen. The results further showed that recombinant *Salmonella enterica* serovar Typhi expressing HIV-1 Gag expressed from the chromosome could be immunogenic. This further gives *Salmonella* the potential to deliver HIV antigens to the immune system.

Codon optimization of expressed HIV-1 *gag* gene can have an impact on the successful delivery of the antigen by the *Salmonella* vaccine vectors. It is therefore critical to consider codon optimization of HIV-1 *gag* for optimal and stable expression in *Salmonella* vaccine vectors and if a strong immune response is to be induced. In our previous study mentioned above, we codon optimized the HIV-1 *gag* and expression of the antigen was shown to be improved in *Salmonella* [88]. In our earlier study, we had shown that a recombinant *Salmonella* expressing wild-type HIV-1 *gag* gene (not codon optimized) was poorly immunogenic in vaccinated mice (unpublished data). Therefore, codon optimization of genes for expression in recombinant *Salmonella* vaccine vectors seems to have an impact on the nature, breadth, and magnitude of the immune responses induced after vaccination. Improved antigen-specific immune responses against a *Salmonella*-based vaccine expressing human papillomavirus type 16 L1 after codon-optimization has been demonstrated [93]. Expression of measles virus (MV) epitopes in *Salmonella* vaccine vector was also enhanced by codon optimization [94]. Oral vaccination of mice with the recombinant *Salmonella* vector induced MV-specific serum antibodies and CD4^+^ T cell response [94]. In another study, it was also shown that an attenuated *Salmonella* vaccine expressing codon optimized HIV-1 Gag was efficient in inducing Gag-specific mucosal IgA and CD8^+^ T cell responses in intestinal lymphoid tissues of orally vaccinated mice [95]. Therefore, in developing recombinant *Salmonella* vaccines for HIV-1, it is critical to optimize the viral genes for expression by the vector as this improves the nature, quality, and magnitude of the immune responses elicited.

## 7. *Salmonella* Vaccine Vectors Delivering HIV Gag DNA Vaccines

The feasibility of using recombinant *Salmonella* to deliver plasmid DNA vaccines to the immunological inductive sites of the mucosal surfaces has, therefore, already been established [96]. Naked DNA vaccines on their own have been used for induction of potent immune responses, especially cell-mediated [97,98]. In more recent years, the use of attenuated *Salmonella* vaccines as delivery vectors for these DNA vaccines has been explored [99,100,101,102]. The actual mechanisms by which *Salmonella* deliver DNA vaccine to elicit immune responses are not yet clear. It has, however, been hypothesized that the DNA vaccine is first delivered specifically into antigen-presenting cells such as macrophages and dendritic cells, which can then express, process and present the antigen peptides for induction of an immune response [96]. It has been demonstrated that recombinant *Salmonella enterica* serovar Typhimurium vaccines carrying DNA vaccines can be delivered *in vivo* to host cells such as macrophages [103,104,105,106,107]. In one study, *Salmonella* carrying an HIV-1 Gag (p24) DNA vaccine was shown to induce HIV-specific immune responses in vaccinated mice [90]. However, MCP3 was used as an adjuvant for the *Salmonella* carrying HIV-1 Gag DNA plasmid [90]. As noted by other studies, co-delivery of DNA vaccines expressing cytokine genes can enhance immunogenicity of vaccines [108,109]. More work needs to be done to explore the potential of using *Salmonella* vaccines as delivery vectors for HIV-1 Gag DNA vaccines. 

## 8. Opportunities for the Future

Since there is growing evidence that attenuated *Salmonella* bacterial vaccines can be used as antigen delivery vectors, future studies should continue to explore new possibilities. Other approaches of delivery of HIV-1 antigens by *Salmonella* vaccines should be investigated in the future. These approaches include the expression and surface display of HIV-1 Gag antigens by the *Salmonella* vectors. Naturally existing *Salmonella* proteins or appendages, such as fimbriae and flagellin, may be used to display HIV-1 Gag antigens on the surface of *Salmonella* vectors. The HIV-1 Gag may be fused to genes of these appendages or outer membrane proteins such as OmpC of *E. coli*, OmpB of *Vibrio cholerae* or OprI of *Pseudomonas aeruginosa* in an expression plasmid. Expression of HIV-1 Gag in different extra-cytoplasmic compartments (in the periplasm, outer membrane, or extracellularly of *Salmonella* vectors may also need future investigations. The sub-cellular localization of the HIV-1 Gag antigens in *Salmonella* vectors is anticipated to influence the nature and magnitude of immune responses elicited after vaccination(s). Future studies should also explore the use *in vivo* inducible promoters since use of constitutive promoters to express HIV-1 antigens normally causes metabolic burden. The NirB promoter from the anaerobically inducible nitrite reductase operon of *E. coli* can be used for expression of HIV-1 Gag in *Salmonella* vectors. NirB promoter is only activated when the *Salmonella* is in the oxygen-deficient environment of the macrophages. The induction of the promoter, only inside the macrophage, prevents loss of the expression plasmid during infection. The NirB promoter has also been successfully used in the expression of other foreign antigens to be delivered by *Salmonella* vaccines [110,111]. Other promoters that can be investigated for use in the *in vivo* expression of HIV-1 antigens are the htrA, groEL, PgaC, and other *Salmonella*-SPI2-derived promoters, which are activated after uptake of the recombinant bacteria by antigen-presenting cells [112,113,114]. It has been concluded that the *in vivo* inducible promoters could improve vaccine stability and immunogenicity than the constitutive promoters [115]. An alternative strategy to genetic stability of recombinant *Salmonella* vectors, which needs future studies, is the use of balanced-lethal plasmid stabilization systems such as the use of *asd* gene. In *asd* *Salmonella* mutants, the loss of the plasmid carrying the *asd* gene *in vivo* is lethal, and only *Salmonella* harboring the recombinant plasmid survive [116,117]. Using this system, plasmid instability normally associated with HIV-1 Gag expression in *Salmonella* vaccine vectors may be circumvented. Co-delivering genes for cytokines and co-stimulatory molecules with HIV-1 genes also provides a possibility of fine-tuning the magnitude and direction of the immune responses induced. Future studies should investigate this possibility of modulating the host immune system against HIV-1 Gag antigens by the production of *in vivo* functional cytokines by *Salmonella* vaccine vectors. Biologically-active cytokines can easily be co-delivered by *Salmonella* [118,119]. It has already been demonstrated that recombinant *Salmonella* co-expressing IL-2, IFN-γ, and macrophage migration inhibitory factor with recombinant antigen could afford to protect mice challenged with *Leishmania major* [120]. The field of using *Salmonella* to deliver HIV-1 antigens such as Gag is therefore promising and needs further exploration.

## 9. Conclusions

The search for candidate HIV-1 Gag-based vaccines continues unabated despite major scientific hurdles in the field. The use of live attenuated *Salmonella* bacterial vaccine vectors is one of the most promising and pragmatic strategies in the development of such HIV-1 Gag vaccines. These vectors have several advantages including the capability of stimulating both mucosal and systemic arms of the immune system.
